# Supplementary material for: Mapping the evolution of fertility support policies in China: A content and instrumental analysis
Source: PLoS One. 2025 Oct 9;20(10):e0332137. doi: 10.1371/journal.pone.0332137 (PMC12510515; doi:10.1371/journal.pone.0332137)
Supplement: S1 Appendix — (ZIP) [file pone.0332137.s001.zip › S1 Appendix. 226 original policy documents/38-国家卫生健康委办公厅关于完善生育登记制度的指导意见(FBM-CLI-4-5112749).doc]

国家卫生健康委办公厅关于完善生育登记制度的指导意见

**发布部门：** [国家卫生健康委员会](https://www.pkulaw.com/law/chl?Aggs.IssueDepartment=60321) [机构沿革](javascript:void(0))

**发文字号：**国卫办人口发〔2021〕21号

**发布日期：**2021.12.09

**实施日期：**2021.12.09

**时效性：** [现行有效](https://www.pkulaw.com/law/chl?Aggs.TimelinessDic=01)

**效力级别：** [部门规范性文件](https://www.pkulaw.com/law/chl?Aggs.EffectivenessDic=XE0302)

**法规类别：** [计划生育管理](https://www.pkulaw.com/law/chl?Aggs.Category=09705)

国家卫生健康委办公厅关于完善生育登记制度的指导意见
（国卫办人口发〔2021〕21号）

各省、自治区、直辖市及新疆生产建设兵团卫生健康委：
　　完善生育登记制度，是实施好三孩生育政策及配套支持措施的内在要求，是完善国家生命登记管理制度、健全覆盖全人群和全生命周期的人口监测体系的重要举措，是强化人口服务体系建设、提升基层服务管理水平的基础性工作。根据优化生育政策工作总体要求，现就完善生育登记制度提出以下意见。
　　**一、**总体要求
　　深入贯彻《中共中央国务院[关于优化生育政策促进人口长期均衡发展的决定](https://www.pkulaw.com/chl/1247bc81dccaeed3bdfb.html)》和《[中华人民共和国人口与计划生育法](https://www.pkulaw.com/chl/d560ce000fba464cbdfb.html)》，坚持以人民为中心，本着依法行政、便民高效、服务引领、信息支撑的原则，完善生育登记制度，加强基层服务管理，保障优化生育政策工作，推动提升家庭发展能力，促进人口长期均衡发展。

　　**二、**着力精简登记事项
　　（一）完善登记流程。夫妻生育子女的，实行生育登记。
　　夫妻在生育前进行生育登记，也可在生育后补登。夫妻可在一方户籍地、现居住地的乡镇（街道）卫生健康工作机构办理生育登记。无法亲自办理生育登记的，可委托村居（社区）卫生健康工作人员代为办理。
　　（二）简化登记要求。登记时，夫妻双方一般需提供身份证或户口本等有效证件。能够通过人口健康相关信息系统获取的信息，不再要求申请人提供证明材料；暂时无法获取的，可通过当事人承诺的方式办理。
　　（三）全面推行网上办理。推广电子化登记，各地应普遍实现可通过网上办事大厅、移动客户端等信息平台办理生育登记。强化出生人口信息共享，打通业务链条和数据共享堵点，实现“数据多跑路、群众少跑腿”，推动网上办、掌上办、一次办。

　　**三、**改进和加强相关服务
　　（一）优化生育服务。充分发挥生育登记的信息引导作用，动态掌握群众生育状况及服务需求，及时提供生育咨询指导、增补叶酸、孕前优生健康检查、孕产妇健康管理、预防接种、婴幼儿照护、儿童健康管理等相关服务。
　　（二）方便群众办事。结合实施三孩生育政策，改进办事流程，加强政策宣传和解释，方便群众办理生育保险等事项。坚持简政便民原则，积极协调相关部门，推动相关事项办理与生育登记脱钩。
　　（三）推进“出生一件事”联办。完善国家生命登记管理制度，加强部门协同，大力推进出生医学证明、儿童预防接种、户口登记、医保参保、社保卡申领等多个事项联办。
　　（四）做好人口监测。把生育登记和出生信息统计作为出生人口监测的重要途径，推动健全覆盖全人群和全生命周期的人口监测体系，密切监测生育形势和人口变动趋势，为实施三孩生育政策及配套支持措施提供信息支撑。

　　**四、**工作要求
　　（一）各级卫生健康部门要高度重视完善生育登记制度工作，将其作为优化生育政策和“我为群众办实事”的一项重要内容，加强组织领导，结合本地实际推进改革创新，提高时效性和准确性。
　　（二）优化整合卫生健康服务资源，加强基层网络和队伍建设，加强宣传培训，做好政策衔接，提高工作效率，保障群众合法权益，最大程度便民利民。
　　（三）加强对生育登记制度落实情况的监督指导，及时总结推广好经验、好做法、好典型；对工作不力、敷衍塞责的，要责令限期整改。
　　自本指导意见印发之日起，《[国家卫生计生委办公厅关于做好生育登记服务工作的指导意见](https://www.pkulaw.com/chl/db8e3faf7b8666d1bdfb.html)》（国卫办指导发﹝2016﹞20号）即行废止。

国家卫生健康委办公厅
　　2021年12月9日

© 北大法宝：（[www.pkulaw.com](https://www.pkulaw.com/)) 专业提供法律信息、法学知识和法律软件领域各类解决方案。北大法宝为您提供丰富的参考资料，正式引用法规条文时请与标准文本核对。
欢迎查看所有[产品和服务](https://www.pkulaw.com/helps/2.html)。法宝快讯：[如何快速找到您需要的检索结果？](https://www.pkulaw.com/helps/70.html)[法宝](https://www.pkulaw.com/helps/69.html)V6有何新特色？

扫描二维码阅读原文

**原文链接：**<https://www.pkulaw.com/chl/7a593921527f5e1cbdfb.html>
